# Factors associated with age of presentation of pediatric feeding disorder

**DOI:** 10.1002/brb3.3461

**Published:** 2024-03-11

**Authors:** Tut Galai, Gal Friedman, Nataly Kalmintzky, Kim Shemer, Dana L Gal, Shlomi Cohen, Hadar Moran‐Lev

**Affiliations:** ^1^ Pediatric Gastroenterology Dana Dwek Children's Hospital, Affiliated to the Faculty of Medicine Tel Aviv Israel; ^2^ Pediatrics Dana Dwek Children's Hospital, Affiliated to the Faculty of Medicine, Tel Aviv University Tel Aviv Israel

**Keywords:** age, pediatric feeding disorder

## Abstract

**Aim:**

Understanding the association between pediatric feeding disorder (PFD) and age of presentation is limited. We aimed to investigate factors associated with PFD among different age groups.

**Methods:**

Retrospective analysis of medical records of infants and toddlers diagnosed with PFD, according to the World Health Organization‐based definition. We compared children aged 1–12 months to those aged 13–72 months.

**Results:**

Included were 253 children with PFD (median [interquartile range] age 16.4 [9.5–33] months at diagnosis, 56% boys). Significantly more children in the younger age group were girls (52.6% vs. 34.4%, respectively, *p* = .03) and preterm (25% vs. 14%, *p* = .03). They had more hospitalizations (34% vs. 23%, *p* = .03) and needed more prescription medications (36% vs. 17%, *p* < .01). Additionally, disturbances in oral intake were primarily linked to feeding skills dysfunction in the younger group and nutritional dysfunction in the older group (39.6% vs. 23.7% and 55% vs. 38%, respectively, *p* = .02).

**Conclusions:**

Infants under 1 year old with PFD represent a distinct patient group with unique characteristics and outcomes. The age of presentation plays a significant role in children with PFD, necessitating tailored treatment strategies.

## Graphical abstract

This is a retrospective analysis of medical records of infants and toddlers diagnosed with pediatric feeding disorder (PFD), according to the World Health Organization‐based definition. Significantly more children in the younger age group (1–12 months) were born preterm. They had more hospitalizations and needed more prescription medications. Additionally, disturbances in oral intake were primarily linked to feeding skills dysfunction in the younger group and nutritional dysfunction in the older group (13–72 months). Further longitudinal studies are needed in order to better assess the outcome of specific therapeutic modalities among children of different ages who are diagnosed with PFD.

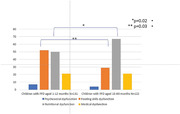

## 1 INTRODUCTION

Pediatric feeding disorders (PFDs) comprise a heterogeneous group of conditions with no universally accepted classification. They may include but are not limited to poor appetite, food selectivity, food refusal, and delayed or absent development of feeding skills, which may or may not be accompanied by inappropriate growth (Bryant‐Waugh et al., 2010). PFD is a relatively common clinical diagnosis with increasing prevalence (Manikam & Perman, 2000; Wright et al., 2007; Kovacic et al., 2021). According to Wright et al. (2007) up to 20% of parents are reportedly concerned about their child's feeding behavior, resulting in considerable numbers who seek professional help.

Feeding disorders in the pediatric population had been historically divided between organic and nonorganic types based upon medical or psychological/behavioral problems, respectively (Bithoney et al., 1989; Budd et al., 1992) The International Statistical Classification of Diseases and Related Health Problems, 10th Revision (ICD‐10) for PFD requires the absence of organic disease (Organization, 1993). The Diagnostic and Statistical Manual of Mental Disorders, 5th (DSM‐V) Edition diagnosis of avoidant/restrictive food intake disorder incorporates nutritional complications and acknowledges that feeding disorders can interfere with psychosocial functioning as well as being common in certain medical conditions (Attia et al., 2013). The DSM‐V diagnosis, however, requires that the priority of the eating disturbance exceeds that associated with the underlying condition and specifically excludes children whose primary challenge is a feeding skill deficit. As the etiology of PFD is often multifactorial and involves the disruption of more than one system, a multidisciplinary approach to diagnosis and intervention appears to be optimal for better identifying and treating the underlying causes.

A unifying diagnostic definition of PFD was recently proposed by a panel of experts in the care of children with feeding disorders based upon the framework of the World Health Organization (WHO) International Classification of Functioning, Disability and Health (Goday et al., 2019; World Health Organization [WHO], 2001) and was accepted as part of the ICD diagnostic nomenclature as of October 2021 (ICD‐10‐CM R63.31). That WHO framework defines “functioning” as an umbrella term referring to all body functions and “disability” as an umbrella term covering impairment (a problem in body function or structure), activity limitation (difficulty encountered in executing a task or action), and restricted participation (problems in involvement in life situations) (WHO, 2001). According to that definition, PFD comprises impaired oral intake that is not age‐appropriate and one that is associated with medical (anatomical, cardiorespiratory, gastrointestinal, and neurological impairments), nutritional (restricted quality, quantity, or diversity of food which may result in malnutrition or micronutrient deficiency), feeding skill (need for texture, feeding position, equipment, or feeding strategies modification), and/or psychosocial dysfunction (inappropriate caregiver management of child's feeding and/or nutrition needs). This classification system describes the impact of PFD on a child's physical, social, emotional, and cognitive functions, as well as on the caregiver‐child relationship, and allows better characterization of heterogeneous populations in order to include all relevant disciplines in the treatment protocol (Goday et al., 2019).

Factors associated with the child, the caregiver, and the feeding environment can adversely affect the acquisition of feeding skills and ultimately contribute to and maintain PFD (Berlin et al., 2011; Burklow et al., 1998). We had recently shown that low socioeconomic status (SES), absence of breastfeeding, and low birth weight (BW) were significantly more frequent in children with PFD (Galai et al., 2022). However, factors associated with age of presentation of feeding disorder were not assessed in details.

A child's age plays a crucial role in understanding and addressing feeding developmental milestones and potential feeding disorders. The literature suggests that there may be critical periods in the development of feeding skills, such as complementary and lumpy solid food introduction (Coulthard et al., 2009; Fewtrell et al., 2017; Northstone et al., 2001) or self‐feeding (Kerzner et al., 2015), and that delays in feeding milestones may lead to the development of later feeding difficulties (Sdravou et al., 2023).

Recent studies have evaluated PFD among different age groups. Kovacic et al., 2021 demonstrated an increasing prevalence of PFD among children in all age groups in the US, with the highest prevalence in children under 5 years of age (Kovacic et al., 2021). Bertrand et al. (2022) analyzed eating profiles among children according to four age groups. However, there are sparse data on the critical time period between 1 month and 2 years of age (Estrem et al., 2022), during which children develop feeding skills and learn about food variety and meal structure. In addition, there is limited information about the effect of the age of presentation of feeding disorders and their patterns. We conducted this study to fill in the gaps in these data.

## 2 METHODS

### 2.1 Patient population and study design

The sociodemographic and clinical data of all infants and children with PFD aged 0 to 60 months who first presented to the multidisciplinary feeding clinic of the Institute of Pediatric Gastroenterology, Hepatology and Nutrition at Dana‐Dwek Children's Hospital, Tel Aviv Medical Center from January 2018 to January 2020 were collected. Excluded were all children who were seen at the clinic in whom the diagnosis of a PFD was ruled out according to the new consensus definition (see below), as well as any patients with missing essential data. The data of children that were diagnosed in the first year of life as having PFD were compared to these that developed PFD between 13 and 60 months of age. The study protocol was approved by the institutional review board (“Helsinki”) of the medical center (reference number—TLV‐0590‐20). Informed consent of the participants was waived as the data retrieved from the medical records were anonymized. The data were handled in accordance with the Principles of Good Clinical Practice.

### 2.2 Data collection

The Feeding Disorder Clinic team includes a gastroenterologist, a dietitian, speech therapists, and a psychologist. Diagnosis and treatment protocols are identical for all age groups. During the first meeting in the clinic, the entire team is present, anamnesis and anthropometric measures are taken and the feeding/eating pattern is determined. A personalized treatment plan is then provided to the parent, which includes nutritional recommendations and a referral to a speech, occupational, psychological therapy, and a medical workup (outpatient or inpatient) based upon the specific patient's needs and the information retrieved in the meeting. The information in the medical files contains both self‐reported patient/parental information and the team's notes on diagnoses, management, and surveillance. All hospital medical records are electronic, with additional access to the individual's health maintenance organization laboratory data. The information retrieved from the children's medical files included:

1. Sociodemographic characteristics: age at presentation of feeding disorder, sex, home address, country of birth, number of children in the family, parental academic background, and parental marital status.
2. Medical history: perinatal characteristics (pregnancy complications, delivery method, BW, gestational age, and birth complications), medications that were prescribed 1 year before the first clinical encounter, developmental status, background conditions, and hospitalizations 1 year before the first clinical encounter.
3. Feeding history: breastfed or bottle fed and age at introduction of complementary food.

### 2.3 Definition of study variables

The SES was determined by the patient's home address according to the Israel Central Bureau of Statistics’ Characterization and Classification of Statistical Areas within Municipalities and Local Councils by the Socio‐Economic Level of the Population 2015 (Israel Central Bureau of Statistics (CBS), 2019). The SES was scored by clusters of localities of residence ranging from 1 to 10, with 1 being the lowest rating and 10 the highest. The SES index is an adjusted calculation of 14 variables that measure social and economic levels in the domains of demographics, education, standard of living, and employment (ranging from the lowest [−2.797] to the highest [2.590]). Academic background was defined according to the sociodemographic status of parents that completed tertiary education.

Low BW was defined as being below 2500 g. Pregnancy complication was defined as any high‐risk pregnancy due to maternal or fetal problems (e.g., gestational diabetes mellitus, preeclampsia, cholestasis, intrauterine growth retardation, or multiple pregnancy). Delivery complication was defined by the presence of maternal fever, premature rupture of membranes, shoulder dystocia, or perinatal asphyxia.

Medications were defined by any medication that was prescribed to the child during the year before the first clinical encounter. Hospitalization was defined by hospitalization for any reason from birth until the first clinical encounter.

PFD was diagnosed by the clinic's multidisciplinary team and according to the consensus definition of the WHO International Classification of Functioning, Disability and Health as a disturbance in oral intake of nutrients inappropriate for age lasting for at least 2 weeks and an absence of cognitive processes consistent with eating disorders and pattern of oral intake not due to a lack of food or to being incongruent with cultural norms (Goday et al., 2019). The type of the child's feeding disorder was defined by the clinical team based upon the anamnesis of the patients and parents and reports in the medical files. The same team retrospectively reviewed the medical files and, based upon that information, divided the types into a nutritional disorder (any case of malnutrition, specific nutrient deficiency, or reliance upon oral supplements to sustain nutrition), feeding skill dysfunction (use of modified feeding strategies, position, or food texture), medical (any medical conditions that could interfere with normal age‐appropriate eating practice, e.g., cleft palate, absent swallowing reflex, etc.), and psychosocial dysfunction (any case of avoidance behaviors when being fed or inappropriate caregiver management of the child's feeding). The code of the predominant type (nutritional, feeding skill, medical, and psychosocial dysfunction) was used in cases of an overlap of PFD manifestations. There was disagreement between the team in fewer than 10% of the cases, and in these cases the dominant feeding disorder type was defined as the one that received the most votes.

### 2.4 Statistical analyses

The data were analyzed with the Statistical Package for the Social Sciences software version 27 (SPSS Inc.). All statistical tests were two‐sided. The Kolmogorov–Smirnov and the Shapiro–Wilk test were applied to assess the normality of continuous data. The data are expressed as means ± standard deviation for normally distributed variables and median and interquartile range (IQR) for skewed distributions. Pearson's chi‐square or Fisher's exact test was performed to compare the distribution of categorical variables between children that were diagnosed in the first year of life as having PFD and children that developed PFD between 13 and 60 months of age. An independent sample *t*‐test or an independent sample Mann–Whitney was performed to compare between children that were diagnosed in the first year of life as having PFD compare to these that was diagnosed between 13 and 60 months for continuous variables with a normal or skewed distribution, as appropriate. The Kruskal–Wallis test was used to compare four types of feeding disorders according to age of presentation of PFD. A *p* value < .05 was considered significant.

## 3 RESULTS

### 3.1 Study population

Table 1 lists the demographic and clinical data of the infants and toddlers with PFD. The cohort comprised 253 patients of whom 142 (56%) were males and who had a median age of 16 [IQR 9.5,33] months at time pf PFD presentation. Excluded were 58 children in whom the diagnosis of a PFD was ruled out as well as 61 patients with missing essential data. According to the consensus PFD definition, the disturbance in oral intake was predominantly related to a nutritional dysfunction in 118 children (46.6%), to feeding skill dysfunction in 82 (32.4%), to a medical condition in 42 (16.6%), and to a psychological dysfunction in 11 (4.3%). Twenty‐one children (8.3%) were treated with cyproheptadine, 45 (17.7%) with proton pump inhibitors, and 12 (4.7%) children received nutritional supplementation via gastrostomy. Seventy‐three children (28.8%) were hospitalized. The main reasons for admission were failure to gain weight in 25 (34.2%), human respiratory syncytial virus (RSV) bronchiolitis and other respiratory complications in 18 (24.6%), and complications of an underlying medical condition in 13 (17.8%).

**TABLE 1:** Demographic and clinical characteristics of children with feeding disorders.

	Feeding disorder group *n* = 253
Sex, male	142 (56%)
Age, months	16 (9.5–33)
Gestational age, weeks	39 (38,40)
Delivery by C/S	45 (17.7)
Birth weight, grams	2866.93 (2489.5, 3363.75)
Breastfeeding	184 (72.7)
Age at complementary food introduction, months	6 (4,6)
Developmental delay	68 (27)
Cyproheptadine treatment	21 (8.3)
Proton pump inhibitors treatment	45 (17.7)
Gastrostomy	12 (4.7)
Background disease	94 (37)
Allergy	21 (8.3)

*Note*: The data are expressed as median and interquartile range for continuous variables and *n* (%) for categorical variables.

Abbreviations: SD, standard deviation; C/S, cesarean section.

## 4 FACTORS ASSOCIATED WITH AGE AT PFD PRESENTATION

A total of 131 (51.7%) children presented with PFD at 1–12 months of age and 122 (48.2%) at 13–60 months of age. There were significant perinatal and clinical differences between those two groups of children. More children in the former group were girls (69 [52.6%] vs. 42 [34.4%] in the latter group, *p* = .03, Table 2) and preterm (33 [25.1%] vs. 18 [14.7%], *p* = .03). The younger children with PFD also had higher rate of hospitalizations (45 [34.3%] vs. 28 [22.9%,], *p* = .03). The main reasons for hospitalization in the younger age group were failure to gain weight in 16 children (35.5%), RSV bronchiolitis in 10 (22.2%), acute gastroenteritis with dehydration in 8 (17.7%), and complications of an underlying medical condition in 7 (15.5%). Among the older age group, the main reasons for hospitalization were failure to gain weight in 9 (32.1%), bronchiolitis and other respiratory complications in 8 (28.5%), complications of an underlying medical condition in 6 (21.4%), and fever in 4 (14.2%). In addition, more children in the younger group were treated with prescription medications during the year before the first clinical encounter (47 [35.8%] vs. 21 [17.2%], *p *< .01) (Table 2).

**TABLE 2:** Demographic and clinical characteristics of children with pediatric feeding disorder (PFD) in two age groups.

	Children with PFD aged 1–12 months *n* = 131	Children with PFD aged 13–60 months *n* = 122	*p* Value
Sex, male	62 (47.3)	80 (65.5)	**.03**
Age, months	10 (6,12)	30.5 (20.75,56.25)	**<.001**
Socioeconomic status Cluster, mean ± SD Index, mean ± SD	7 (6,8) 1.05 (.2,1.2)	7 (7,8) 1.05 (.2, 1.2)	.84 .77
Parents’ marital status Married Divorced Single parent	36 (27) 5 (3.8) 6 (4.5)	30 (24) 1(.8) 9(7.3)	.4
Parental tertiary academic background	96 (73)	89 (72)	.4
Number of siblings	1 (1,2)	1 (1,2)	.2
Delivery complications	13 (9.9)	7 (5.7)	.1
Low birth weight	37 (28.2)	30 (24.5)	.2
Delivery by C/S	18 (13.7)	19(15.5)	.6
Preterm	33 (25.1)	18 (14.7)	**.03**
Birth weight, grams	2900 (2387.5,3300)	3037.5 (2541.25,3500)	.07
Breastfeeding	98 (74.8)	86 (70.4)	.3
Age at complementary food introduction, months	6 (5,6)	6 (4,6)	.75
Developmental delay	40 (30.5)	45 (36.8)	.2
Medication	47(35.8)	21 (17.2)	**<.01**
Hospitalization	45 (34.3)	28 (22.9)	**.03**
Gastrostomy tube feeding	6 (4.5)	6 (4.9%)	.5
Background disease	49 (37.4)	45 (36.8)	.5
Allergy	10(7.6)	11 (9)	.4

*Note*: The data are expressed as median and interquartile range for continuous variables and *n* (%) for categorical variables. **Bold** indicates significant.

Abbreviations: SD, standard deviation; IUGR, intrauterine growth restriction; *C/S*, cesarean section.

**FIGURE 1:**
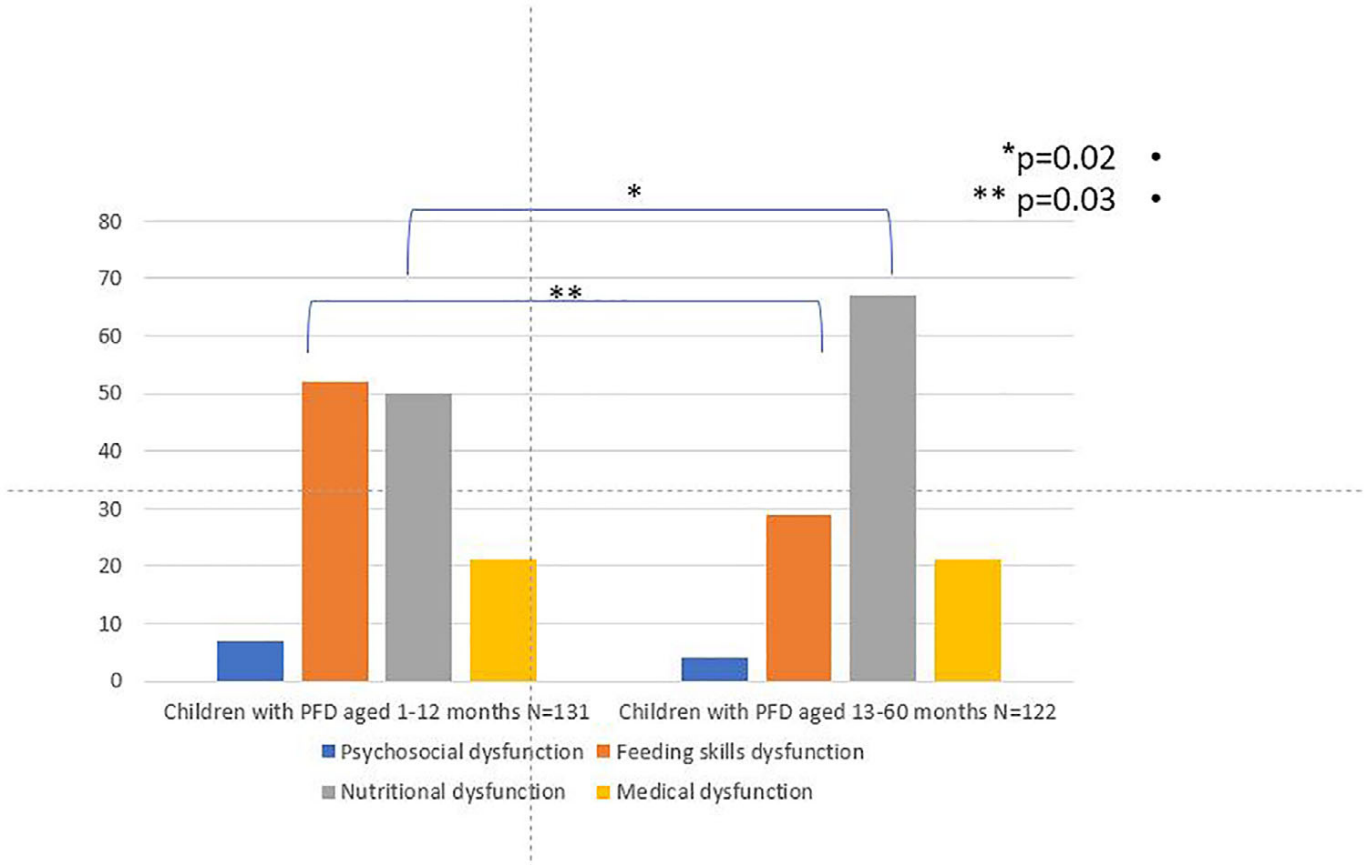
Pediatric feeding disorder types in children ≤1 year and >1 year of age according to the World Health Organization (WHO) consensus group definitions.

There were also PFD subtype differences between the age groups. According to the WHO‐based definition, impaired oral intake was predominantly related to feeding skill dysfunction among children below 1 year of age compared with children above 1 year of age (52 [39.6%] vs. 29 [23.7%], respectively, *p* = .02) and to nutritional dysfunction among the older children compared with the younger ones (67 [55%] vs. 50 [38%], respectively, *p* = .03 (Figure 1). There were no group differences in sex, gestational age, low BW, breastfeeding or bottle feeding, age of complementary food introduction, background diseases, documented allergy to food, SES, or marital status of the parents.

## 5 DISCUSSIONS

We investigated characteristics of children with PFD according to their age of PFD presentation. We applied the recently proposed diagnostic term ‘‘PFD’’ for unifying the medical, nutritional, feeding skills, and psychological concerns associated with the disorder. Our results demonstrated clinical differences between children who were diagnosed with PFD when they were below 1 year of age and those who were diagnosed between 1 and 5 years of age. The former children had more hospitalizations and more prescription medications, and more of them were girls and preterm. Moreover, they had significantly higher percentages of PFD related to a feeding skill disorder compared to children with PFD aged 1–5 years whose PFD was predominantly related to nutritional disorders.

Specific feeding problems are often related to the child's age, with typical onset between the ages of 6 months and 4 years (Aldridge et al., 2010). Recent studies have shown an increasing prevalence of PFD among very young children (Kovacic et al., 2021; Lamm et al., 2023), but there is limited information about the characteristics and differences related to age at PFD presentation.

We found higher percentages of prematurity among our younger age group (0–12 months). Earlier studies have shown that feeding difficulties are more prevalent in young children born prematurely (Pados et al., 2021), including our recent study which found more preterm infants among children with a PFD diagnosis compared with non‐preterm controls (Galai et al., 2022). However, the effect of age at feeding disorder presentation among preterm infants had not been previously clarified. One meta‐analysis found that problematic feeding is highly prevalent in prematurely born children during their first 4 years of life, without any difference in the prevalence of problematic feeding by the child's age (Pados et al., 2021). Park et al. (2019) found that prematurely born children aged 6–15 months had significantly more feeding problems than those aged 15 months to 2.5 years, possibly due to the challenging period of the acquisition of feeding skills.

We also found that children below 1 year of age had more hospitalizations compared with our older age group, with the main reasons for hospitalizations being failure to gain weight, RSV bronchiolitis, and acute gastroenteritis with dehydration. Given that sucking and feeding are important early neurodevelopmental milestones, not reaching them may result in hospitalizations for intravenously administered fluids in the case of dehydration or for enteral feeding for a variety of reasons. In addition, any infectious disease may put these children at higher risk for complications, such as dehydration or malnutrition. A large cohort study from England found relatively more hospital admissions during the first year of life and specifically in the early neonatal period, the great majority being accounted for jaundice and feeding difficulties (Jones et al., 2018). The authors of that report assumed that the cause may be related to feeding practice and insufficient support after hospital discharge and that an intensive treatment intervention and the provision of guidance and support during the first year of life may prevent some of these hospitalizations.

The reason that more children in the younger age group were treated with prescription medications during the year before the first clinical encounter presentation compared to the older age group may be related to higher percentages of proton pump inhibitor prescriptions for gastroesophageal reflux disease (GERD) symptoms. The relationship between GERD and feeding disorders in infants had already been described more than two decades ago (Mathisen et al., 1999). Feeding problems affecting behavior, swallowing, food intake, and mother–child interactions are not uncommon in infants with GERD, and the optimal management of these early feeding problems could include a multidisciplinary approach as an adjunct to drug therapy.

Lastly, we found that impaired oral intake among the children in our younger age group was predominantly related to feeding skill dysfunction. This finding is in line with those of Rommel et al. (2003) who reported that oral feeding disorders (mainly sucking and oral sensory) were more common in children younger than 2 years of age. There are some potential explanations for these findings. First, certain developmental stages of infancy and early childhood, which are directly related to stages of adaptations in feeding style and dietary intake (including the weaning, self‐feeding, and mobilization stages), occur during this time (Birch, 1999), and a failure to achieve these milestones may result in feeding skill dysfunction. For example, the transitioning from a solely liquid diet to the introduction of solid foods with various textures during the first year of life can represent one of the initial obstacles to achieving normal and healthy feeding habits. Difficulties during this phase are mainly attributed to the physical and behavioral adjustments required for this developmental milestone. Second, the critical developmental process of acquisition of oral feeding skills occurs by full‐term gestation and may be interrupted by preterm birth or by other comorbidities (Viswanathan & Jadcherla, 2020). Interestingly, in the present study, we found higher percentage of preterm babies in the PFD that presented over the first year and this group was associated with impaired oral intake. Due to improved survival associated with advances in perinatal and neonatal care over the past decade, the prevalence of oral feeding difficulties in infants has increased in parallel over the past decade (Horton et al., 2018). Infants who have limited experience in orally manipulating substances often face difficulties when later confronted with solid foods and different textures. Developing improved oral motor control and decreased sensitivity to touch in the mouth and lips becomes crucial for effectively handling and accepting these new food types (Aldridge et al., 2010). It is therefore important to understand the functional maturity level and the pathophysiological mechanisms behind the specific oral feeding difficulty of each patient in order to provide personalized management strategies for optimizing feeding outcomes.

Impaired oral intake among the children in our older age group was predominantly related to nutritional dysfunction. This may be explained by the higher prevalence of selective eating in this age group, which is characterized by a reluctance to consume familiar foods or explore new ones, accompanied by strong food pf. This behavior can result in a limited range of foods in a child's diet during his/her first years of life. Consequently, there may be concerns about the nutritional balance of their diet, potentially leading to adverse health outcomes (Taylor et al., 2015). Our findings are in agreement with other studies that reported a prevalence of selective eating according to age. Mascola et al. (2010) found an increase in selective eating from 2 years of age that peaked at 6 years of age when the prevalence plateaued. Hafstad et al. (2013) reported the highest prevalence of selective eating at age 3.5 years, and Cardona Cano et al. (2015) observed the prevalence to be highest at 3 years of age compared with ages 1.5 and 6 years.

Several studies support the benefit of a multidisciplinary treatment in PFDs in terms of improving mealtime behaviors, enhancing dietary diversity, assuring a nutritionally complete diet, increasing oral caloric intake, and targeting skill‐based deficits (Kim et al., 2021; Sharp et al., 2017; Volkert et al., 2021). However, there is lack of data on how age can impact treatment type, intensity, and outcome.

This is one of the first studies to provide clinically based evidence for the association between PFD and age of the child at first PFD presentation according to the recent consensus WHO‐based definition. This study is limited by its retrospective nature and the relatively small sample number. Moreover, some degree of incompatibility between clinical diagnosis and the formal new definition of PFD may be anticipated. However, as the diagnosis of PFD was made by a multidisciplinary team comprised of a pediatric gastroenterologist, dietician, speech therapist, and psychologist, we believe that inaccuracies in the diagnosis of PFD and its subtypes were held to a minimum. Lastly, the patient cohort accessed care at our subspecialty clinic, and they may not represent the broader pediatric population. However, our hospital is a tertiary care medical center, which we believe does represent the general pediatric population nationwide.

In conclusion, the findings of our study demonstrate that age at presentation PFD emerges as a significant factor in determining the patterns of PFD. Treatment planning should be based upon the patient's age. Specifically, the focus in the younger age group should be upon the development of feeding skills. Interventions may involve sensory integration therapy, oral motor exercise, self‐feeding encouragement, and the gradual introduction of a variety of textures and tastes. The focus in the older age group should be upon nutritional assessment and recommendations by a specialist dietician. Further longitudinal studies are needed in order to better assess the outcome of specific therapeutic modalities in terms of their level of intensity and their duration among children of different ages who are diagnosed with PFD.

## AUTHOR CONTRIBUTIONS

Hadar Moran‐Lev and Tut Galai conceptualized and designed the study, drafted the initial manuscript, and reviewed and revised the manuscript. Shlomi Cohen designed the data collection instruments, collected data, carried out the initial analyses, and reviewed and revised the manuscript. Kim Shemer, Nataly Kalmintzky, and Dana L Gal conceptualized and designed the study, coordinated and supervised data collection, and critically reviewed the manuscript for important intellectual content. All authors approved the final manuscript as submitted and agree to be accountable for all aspects of the work.

## CONFLICT OF INTEREST STATEMENT

The authors declare that they have no conflicts of interest.

## FUNDING INFORMATION

The project received no specific support.

### PEER REVIEW

The peer review history for this article is available at https://publons.com/publon/10.1002/brb3.3461.

## Data Availability

The datasets used and/or analyzed during the current study are available from the corresponding author on reasonable request.
